# Case Report: Misdiagnosis of a lipofibromatosis-like neural tumor of the dorsal skin as dermatofibrosarcoma protuberans

**DOI:** 10.3389/fsurg.2024.1417263

**Published:** 2024-09-13

**Authors:** Xiaowei Zhang, Chen Yan, Tingting Xu, Jiajia Ying

**Affiliations:** ^1^ Department of Pathology, Affiliated Dongyang Hospital of Wenzhou Medical University, Dongyang, Zhejiang, China; ^2^ Department of Surgical Center, Affiliated Dongyang Hospital of Wenzhou Medical University, Dongyang, Zhejiang, China

**Keywords:** skin, soft tissue tumor, lipofibromatosis-like neural tumor, misdiagnosis, case report

## Abstract

**Background:**

Lipofibromatosis-like neural tumors (LPF-NT), which have only recently been established, are intermediate soft tissue tumors with neurotrophic tropomyosin receptor kinase 1 (*NTRK1)* gene alterations and are typically misdiagnosed as dermatofibrosarcoma protuberans, low-grade malignant peripheral nerve sheath tumors, or spindle cell lipoma due to their histopathological and immunohistochemical expression of CD34 and S-100.

**Case presentation:**

The patient was admitted to our hospital with a painless back mass that had appeared more than 4 years prior to admission. Physical examination revealed a subcutaneous mass on the back, approximately 1.5 cm in diameter and protruding into the skin, with clear boundaries and no tenderness. The tumor was surgically resected. The postoperative pathological results suggested a spindle cell soft tissue tumor, and dermatofibrosarcoma protuberan was initially considered. After consultation at a provincial hospital, the patient was diagnosed with a cutaneous lipofibromatosis-like neural tumor of the back. A second extended resection was then performed. Intraoperative rapid freezing examination revealed negative incision margins.

**Conclusion:**

Histological and immunohistochemical detections aid in the differential diagnosis of LPF-NTs. Complete surgical resection is the preferred treatment for LPF-NTs.

## 1 Introduction

Lipofibromatosis-like neural tumors (LPF-NTs) are locally aggressive soft tissue tumors that have only recently been documented. Typically, LPF-NTs exhibit a fascicular arrangement and invasive growth patterns similar to those seen in lipofibromatosis. However, these tumor cells are positive for both S100 and CD34 immunohistochemical markers, and they characteristically exhibit neurotrophic tropomyosin receptor kinase 1 (*NTRK1*) gene rearrangement. This section analyzes the histopathological features of a case of LPF-NT that was initially misdiagnosed as dermatofibrosarcoma protuberans of the skin. Additionally, it reviews relevant literature to discuss the pathogenesis, diagnosis, and differential diagnosis of LPF-NT, aiming to enhance understanding of the disease among clinicians and pathologists.

## 2 Case description

### 2.1 Chief complaints

An unintentionally discovered painless mass on the back, has persisted for more than 4 years.

### 2.2 History of present illness

A 29-year-old female was admitted to the hospital for surgical treatment on June 10, 2023, more than 4 years after finding a painless mass on her back. There was no redness, swelling, pain, skin rupture, tenderness, or any other symptoms of discomfort.

### 2.3 History of past illness

The patient had no previous medical history.

### 2.4 Personal and family history

The patient denied any family history of malignant tumors.

### 2.5 Physical examination upon admission

Physical examination revealed a subcutaneous mass on the right side of the back, approximately 1.5 cm in diameter, protruding into the skin, with a clear boundary and no tenderness.

### 2.6 Laboratory examinations

No abnormalities were detected in routine blood and urine analyses.

### 2.7 Imaging examinations

The patient did not undergo imaging examinations as the tumor was superficial.

## 3 Therapeutic intervention

Surgical treatment was conducted on June 10, 2023, under local anesthesia. During the operation, a subcutaneous mass was completely excised following a pre-marked line after lidocaine injection. The subcutaneous and skin tissues were sutured. The surgery was successful with effective anesthesia, and the intraoperative bleeding volume was approximately 1 ml. A nodular mass measuring 1.4 × 1.3 × 1.0 cm was removed, characterized by a pale section with irregular borders.

## 4 Diagnostic assessment

**Figure 1:**
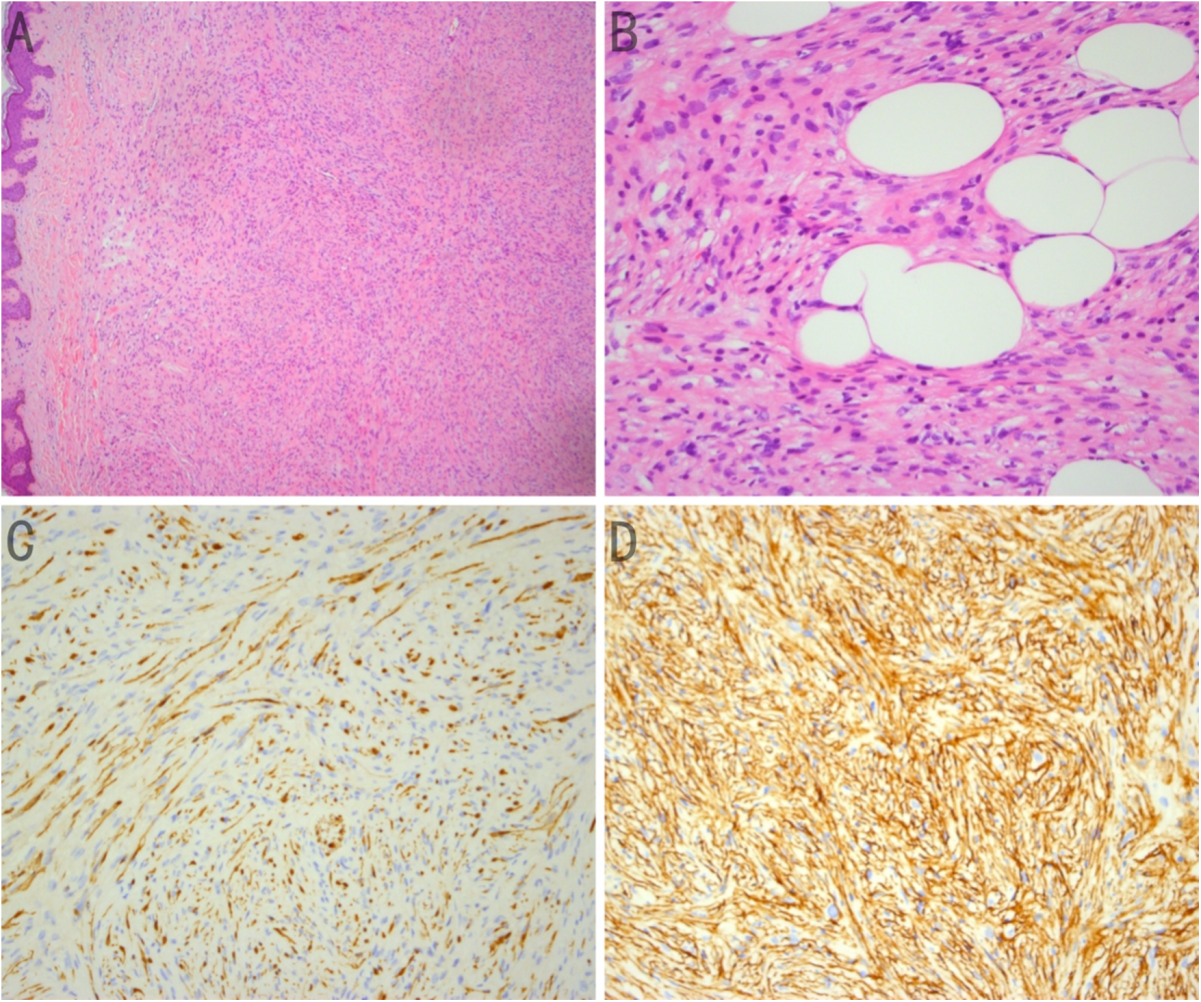
**(A)** Spindle Tumor cells form bundles in the dermis of the skin tissue, similar to those observed in lipofibromatosis; hematoxylin-eosin (HE) staining (×50). **(B)** Spindle tumor cells infiltrating adipocytes; hematoxylin-eosin (HE) staining (×200). **(C)** Strong expression of CD34 in spindle tumor cells; immunohistochemistry (×200). **(D)** Strong expression of S-100 in spindle tumor cells; immunohistochemistry (×200).

Postoperative pathological analysis revealed a skin spindle cell tumor on the back, initially diagnosed as dermatofibrosarcoma protuberans with a positive incision margin; consultation at a higher-level hospital was subsequently recommended. Immunohistochemical staining showed positive results for CD34, Ki-67 (less than 5%), panTRK, SRY-box transcription factor 10 (SOX-10), and S-100, with negative results for CD68, smooth muscle actin (SMA), desmin, CD117, and epithelial membrane antigen (EMA). Based on the pathological histology and immunohistochemical findings of the provincial hospital (Affiliated Shanghai Huashan Hospital of Fudan University), the final diagnosis was confirmed as LPF-NT (back) (Figure 1).

## 5 Outcome and follow-up

After the diagnosis, the patient underwent an extended resection, with a ten-day gap between the initial and subsequent surgeries. Intraoperative rapid freezing diagnosis confirmed negative margins for the peripheral and basal incisions. The wound healed well, with no evidence of recurrences or distant metastases during the initial three months and the subsequent 11 months of follow-up.

## 6 Discussion

Lipofibromatosis is a rare, slow-growing soft tissue tumor composed of adipocytes and fibroblasts (1). LPF-NT, first reported in 2016 by Agaram et al. (2), is a mesenchymal spindle cell soft tissue tumor with a histologic morphology similar to that of lipofibromatosis but with immunohistochemical expression of both CD34 and S100 and often with *NTRK* gene rearrangement. LPF-NT is a new type of soft tissue tumor characterized by fibroblast differentiation, neural differentiation, and *NTRK* gene rearrangement (3). It is rare, and only 73 cases have been reported in the English literature in recent years, most of which were in children (4). LPF-NT is most common in children and young adults but can also occur in middle-aged and older adults (age range, 0–77 years; median age, 13 years), with a slightly higher incidence in men than in women. The clinical manifestations of LPF-NT are non-specific and usually present as painless masses. LPF-NT is most commonly found in the upper or lower extremities, followed by the buttocks, back, head, neck, abdominal wall, and chest. Most cases are limited to subcutaneous tissue and the dermis; however, occasionally, the bone or skeletal muscle is involved.

A previous study on LPF-NT revealed a mass with mild to moderate echo levels in the subcutaneous fat layer and an uneven internal echo using ultrasonography. Moreover, computed tomography and magnetic resonance imaging revealed a localized, low-density, oval mass with varying fat content (5). Histopathologically, the most important differential diagnoses include the following: (A) calcifying aponeurotic fibroma, which is relatively easy to diagnose in typical cases. In the case of early-stage, small or puncture biopsy specimens, the characteristic cartilaginous differentiation nodules and calcification are lacking, adipose tissue is interspersed with spindle cell bundles, and epithelioid fibroblasts are arranged in a cord or radial manner. (B) Dermatofibrosarcoma protuberans is a tumor located in the dermis and extensively involves subcutaneous fat. It is characterized by a consistently fine mat striate structure, short spindle to oval nuclei, light chromatin, and rare stroma lymphocytes, while being CD34-positive and S-100-negative. In addition, (C) low-grade malignant peripheral nerve sheath tumor is characterized by bundle or braided structures rich in cells, alternating between loose and dense tumor regions, at least mild to moderate cell atypia, and active mitosis. Immunohistochemically, the tumor is positive for S100, CD34, and SOX-10 and has an H3K27me3 expression deletion. Furthermore, (D) spindle cell lipoma tumors are typically characterized by a mass with a complete capsular, tumor cells that are fat, short, and spindle-shaped, and stroma rope collagen alongside scattered mast cells. Immunohistochemical expression of CD34 and desmin is commonly detected, whereas retinoblastoma gene (*Rb*) expression is absent. (E) Classic lipofibromatosis typically occurs in children's limbs and is mainly composed of staggered fibrous spindle cells interspersed with adipose tissue. Occasionally, small vacuolar cells can be observed at the junction of spindle cells. Immunohistochemical staining shows positive results for CD34 and SMA and negative results for S100.

The biological behavior of LPF-NT is intermediate and locally aggressive, although it typically does not lead to distant metastasis. Complete surgical resection is the preferred treatment method; if the surgical resection is incomplete, there is a risk of recurrence, and long-term follow-up is necessary after surgery (6). In recent years, successful surgical treatment of large lumbar LPF-NT cases has been reported after the preoperative application of the NTRK inhibitor entitinib. According to a previous study, the *NTRK* gene should be evaluated in all patients, and NTRK inhibitor therapy should be considered for patients unsuitable for tumor resection (7).

Our patient had a dorsal skin tumor over 4 years ago. After the surgical resection of the tumor, the postoperative pathology revealed it as cutaneous dermatofibrosarcoma protuberans, which shares similarities with LPF-NT in terms of pathological histology and immunohistochemistry. LPF-NT is an intermediate soft tissue tumor that has only recently been established; thus, the understanding of the disease among clinicians and pathologists may be insufficient. This tumor type is often misdiagnosed, resulting in incorrect treatment. The diagnosis of LPF-NT depends on pathological examination, and immunohistochemical examination of the co-expression of S100 and CD34 helps identify this type of tumor. The occurrence of *NTRK* gene rearrangement offers a higher diagnostic value (8, 9).

## 7 Conclusion

The histological morphology of LPF-NTs overlaps with that of dermatofibrosarcoma protuberans. The dual expression of S100 and CD34 in immunohistochemistry should raise strong suspicion of lipofibromatosis-like neural tumors, and NTRK gene testing can be a valuable tool for differential diagnosis. Herein, we report a case of an adult LPF-NT that was initially misdiagnosed as dermatofibrosarcoma protuberans of the skin due to the rarity of LPF-NT case reports and limited understanding of its clinicopathological features. Recognizing the clinicopathological features among clinicians and pathologists is pertinent for accurate differential diagnosis.

## Data Availability

The original contributions presented in the study are included in the article/Supplementary Material, further inquiries can be directed to the corresponding author.
